# First Molecular Characterization of Small Ruminant Lentiviruses in Hungarian Goat Population

**DOI:** 10.3390/pathogens13110939

**Published:** 2024-10-29

**Authors:** László Ózsvári, Krisztina Bárdos, Agata Moroz-Fik, Kinga Biernacka, Marcin Mickiewicz, Zofia Nowek, Carlos Eduardo Abril, Giuseppe Bertoni, Snorre Stuen, Saulius Petkevičius, Jarosław Kaba, Michał Czopowicz

**Affiliations:** 1 Department of Veterinary Forensics and Economics, University of Veterinary Medicine Budapest, István u. 2, 1078 Budapest, Hungary; ozsvari.laszlo@univet.hu (L.Ó.); bardos.krisztina@univet.hu (K.B.); 2 National Laboratory of Infectious Animal Diseases, Antimicrobial Resistance, Veterinary Public Health and Food Chain Safety, University of Veterinary Medicine Budapest, 1078 Budapest, Hungary; 3 Division of Veterinary Epidemiology and Economics, Institute of Veterinary Medicine, Warsaw University of Life Sciences-SGGW, Nowoursynowska 159c, 02-776 Warsaw, Poland; agata_moroz@sggw.edu.pl (A.M.-F.); kinga_biernacka@sggw.edu.pl (K.B.); marcin_mickiewicz@sggw.edu.pl (M.M.); nowekz@wp.pl (Z.N.); jaroslaw_kaba@sggw.edu.pl (J.K.); 4 Institute of Virology and Immunology, Department of Infectious Diseases and Pathobiology, Vetsuisse Faculty, University of Bern, Laenggass-Str. 122, CH-3012 Bern, Switzerland; carlos.abril-gaona@unibe.ch (C.E.A.); giuseppe.bertoni@unibe.ch (G.B.); 5 Department of Production Animal Clinical Sciences, Norwegian University of Life Sciences, Svebastadveien 112, N-4325 Sandnes, Norway; snorre.stuen@nmbu.no; 6 Department of Veterinary Pathobiology, Veterinary Academy, Lithuanian University of Health Sciences, Tilzes Str. 18, LT-47181 Kaunas, Lithuania; saulius.petkevicius@lsmu.lt

**Keywords:** CAE, caprine arthritis-encephalitis, real-time PCR, SRLV

## Abstract

In 2023, a molecular study was conducted on the Hungarian goat population to determine genotypes and subtypes of small ruminant lentiviruses (SRLV) infecting these herds. Ten goat herds seropositive for SRLV infection according to a serosurvey conducted earlier in Hungary were selected, and 135 adult goats (>1 year old) were blood sampled. The two-stage nested real-time PCR (nRT-PCR) was used to detect proviral DNA of SRLV and distinguish between two main viral genotypes (A and B). PCR products were submitted for Sanger dideoxy sequencing, and phylogenetic and molecular evolutionary analyses were conducted on the 200–250 bp-long proviral DNA sequences from the end of long terminal repeat (LTR) region and beginning of *gag* gene using the MEGA11 software. Reference strains included strains most identical to Hungarian sequences according to the Standard Nucleotide BLAST and prototypic strains for the relevant genotypes and subtypes. Proviral DNA of SRLV was detected in goats from all ten tested herds. A single SRLV genotype was detected in 6 herds—genotype A in three herds and B also in three herds. In four herds, mixed infection with genotypes A and B was confirmed. In total, 110/135 seropositive goats tested positive in the nRT-PCR (81.5%): 49/110 goats (44.5%) for genotype A, 54/110 goats (49.1%) for genotype B, and 7/110 goats (6.4%) for both genotypes. Hungarian sequences belonged to subtypes A1/A18, A2, and subtype B1. This is the first study which shows that Hungarian goats are infected by SRLV belonging to both genotypes A and B.

## 1. Introduction

Small ruminant lentiviruses (SRLV) are a heterogenous group of retroviruses causing two major infectious diseases of small ruminants—maedi-visna in sheep and caprine arthritis-encephalitis (CAE) in goats. Historically, viruses responsible for these diseases were considered to be two related but different species, named maedi-visna virus (MVV) and caprine arthritis-encephalitis virus (CAEV), respectively [1]. Studies carried out at the end of the 20th century and in the first decade of the 21st century showed that the dichotomous classification of SRLV into “ovine” MVV and “caprine” CAEV was spurious. SRLV freely cross the interspecies barrier between sheep and goats [2,3,4,5,6,7,8] and causes lifelong infections. However, the disease produced is determined by an infected animal species rather than by an infecting virus—in sheep, mainly lungs are affected by interstitial pneumonia, whereas in goats, progressive deforming arthritis develops [9,10]. In goats with CAE, arthritis is typically associated with progressive emaciation and eventually leads to severe lameness, recumbency, and necessitates the culling of affected goats for humane reasons. Rarely can CAE manifest itself with two other clinical syndromes—indurative mastitis and progressive paralysis [10,11,12]. Interstitial pneumonia is also observed in histopathology of the lungs [11,13,14]; however, it has not been described in a clinical form. Nevertheless, the clinical phase of CAE develops in only approximately 20–30% of infected goats [15,16], while the other SRLV-infected goats remain in the subclinical phase until the end of their life. The impact of subclinical disease on goats’ productivity, reproduction, and welfare remains unclear. While most studies have shown various degrees of deterioration of milk yield and quality in goats with subclinical CAE [17,18,19,20,21], no significant negative influence on the number of kids in the litter, birth body weight, and growth of kids from infected dams has been shown [22,23,24].

SRLV turned out to be a complex group of genetic variants whose classification largely depended on the part of the genome analyzed [6,25,26,27,28]. The current classification of SRLV was initially introduced by Shah et al. [29] and then expanded and modified to eventually comprise four genotypes, A, B, C, and E, which include various numbers of genetic subtypes (except for a distinct genotype C restricted to the small ruminant population of Norway) [30,31,32]. Twenty-seven subtypes are now classified in genotype A, five subtypes in genotype B, and two subtypes in genotype E [7,8]. Despite the great genetic diversity of SRLV, the link between genotypes and subtypes of SRLV and their pathogenicity or virulence is still questionable. Some studies have shown increased tropism of particular SLRV strains to certain organs [8,11,33,34,35]. However, more evidence is necessary to prove that such characteristics can be generalized to the subtype as a whole. The only exception is genotype E which appears to be of very low virulence [36,37]. Also, attempts to reveal a clear association between genetic classification and serological response have failed [38,39].

Hungary is a Central European country with a small population of goats; in 2022, approximately 26,000 adult female goats were kept in approximately 1000 herds. Ninety percent of these herds are small (less than 50 adult female goats). Goat farming is mostly extensive, with dairy goat farms producing for local markets and a few larger dairies [40]. When this study was planned within the frame of the CAE-RAPID project financed by the ICRAD Horizon 2020 program in 2020, very little was known about the epidemiological situation of SRLV infection in the small ruminant population of Hungary. In the early 1970s, maedi-visna was introduced into the Hungarian small ruminant population [41] and the small-scale serological studies carried out at the beginning of the 21st century showed that SRLV infection in Hungarian goats was present at the level of 30–50% [42,43]. Moreover, the occurrence of SRLV infection has been reported in goat populations of neighboring countries, such as Romania (seroprevalence ranging from 0.4% to 40%) [44,45,46,47,48,49,50], Serbia [51], and Slovenia [52]. Therefore, we carried out the study to identify genotypes and subtypes of SRLV present in the Hungarian goat population.

## 2. Materials and Methods

### 2.1. Animals and Sampling

The study was carried out in 2023 in 10 Hungarian goat herds in which adult goats (>1 year old) had previously been found to be seropositive for SRLV, using an indirect commercial ELISA, coated with the panel of synthetic peptides from SRLV structural proteins—surface glycoprotein (gp135), transmembrane glycoprotein (gp46), and capsid protein (p25/p28) (ID Screen MVV-CAEV Indirect Screening test, ID.vet Innovative Diagnostics, Grabels, France). This ELISA has been shown to have very high diagnostic sensitivity (dSe) and specificity (dSp) [53,54]. The results of a serological survey preceding this molecular study are presented elsewhere [55,56]. The herds were located in 5 geographic regions of Hungary: Western Transdanubia (4 herds), Northern Great Plain (2 herds), Central Transdanubia, Southern Transdanubia, Southern Great Plain, and Pest (1 herd in each). The herds counted 26 to 182 adult goats with a median (IQR) of 103 (66–126) adult goats, and 1 to 25 males (median of 5) were kept in these herds (Table S1).

In each seropositive herd, a representative sample of seropositive adult goats was randomly selected so that a less common of the two SRLV genotypes (i.e., A and B) could be detected if present in at least 30% of seropositive goats (i.e., expected prevalence of 30%), assuming the dSe and dSp of the two-stage nested real-time PCR (nRT-PCR) of 76% and 100%, respectively [57], and the level of confidence of 95%. The required sample size was 12 goats. If fewer goats in the herd were seropositive, all were included in the study. No ethics approval was required in Hungary for blood collection from farm animals according to the Hungarian legal regulations [58]. The owners of selected herds granted informed consent for participation in the study.

### 2.2. Molecular Testing and LTR-gag Sequencing

Blood samples were collected from the jugular vein in 10 mL tubes with ethylenediaminetetraacetic acid (EDTA) as an anticoagulant (BD Vacutainer, BP-Plymouth, UK) and centrifuged at 3000 rpm (1390× *g*) for 10 min. 500 μL of buffy coat were harvested, and erythrocytes were lysed using 1 mL of the RBC Lysis Buffer (G-Biosciences, St. Louis, MO, USA) to obtain leukocyte pellets, which were then stored at −20 °C until testing. DNA from leukocyte pellets was extracted using the Qiagen DNeasy^®^ Blood and Tissue kit (Qiagen, Hilden, Germany) and eluted in 100 μL of elution buffer according to the manufacturer’s protocol. Extracts were tested using a two-stage nRT-PCR which allowed us to distinguish between proviral DNA of SRLV genotype A and B [57]. Briefly, the nRT-PCR was carried out in two successive amplification steps. The first step was a conventional qualitative PCR and contained 12.5 μL of Hotstar Taq Master Mix (HotStarTaq DNA Polymerase kit, Qiagen GmbH, Hilden, Germany), 0.15 μL of a mixture of 7 primers (Table S2; each at the concentration of 300 nM (nmol/L)), and 5 μL of the extracted DNA. The concentration of extracted DNA in the sample was measured in the NanoDrop One spectrophotometer (ThermoFisher Scientific, Waltham, MA, USA). Amplification started with the activation of the polymerase at 95 °C for 15 min, followed by 40 cycles: 20 s at 94 °C for denaturation of double-stranded DNA, 30 s at 60 °C for annealing of primers to each of the single-stranded original proviral DNA templates, and 45 s at 72 °C for extension (elongation) of the new proviral DNA strands from the primers. In the second step, all products of the first PCR were tested in parallel using two genotype-specific RT-PCRs for a sensitive detection and discrimination between SRLV genotypes A and B. RT-PCR reactions contained 12.5 μL of TaqMan™ Universal PCR Master Mix (Applied Biosystems, Life Technologies, Waltham, MA, USA), 0.25 μL of each primer (Table S2; at the concentration of 900 nM), 0.5 μL of probes (at the concentration of 200 nM), and 5 μL of the PCR product from the first step. Amplification profiles consisted of a hold stage of 20 s at 95 °C and a PCR stage of 45 cycles: 15 s at 95 °C for denaturation and 60 s at 60 °C for annealing. Thermal cycling was performed using the 7300 Real-Time PCR System (Applied Biosystems, Life Technologies, Waltham, MA, USA) and the LightCycler 480 II (Roche Diagnostics, Rotkreuz, Switzerland). Results of nRT-PCR were considered positive if the cycle threshold (Ct) value was below 35. For positive samples, the exact cycle threshold (Ct) values were recorded as a relative information of proviral load in the sample. No reference curve was used, so the results were not quantitative.

From each nRT-PCR-positive herd, one PCR product of each SRLV genotype (A and B) was gel-extracted using primers from the 2nd stage of PCR but without the probes (Table S2). Then, PCR products were sequenced in both directions using the Sanger dideoxy sequencing method in a commercial laboratory (Genomed SA, Warsaw, Poland) using 3730 xl DNA Analyzer (Applied Biosystems, Foster City, CA, USA) and a BigDye Terminator v3.1 Cycle Sequencing kit. The obtained SRLV sequences were manually checked and edited using Geneious Prime software version 2024.0.7 (GraphPad Software, LLC, Boston, MA, USA). The genetic relatedness among the SRLV strains was analyzed based on approximately 200–250 bp-long fragments covering mainly the long terminal repeat (LTR) and the beginning of the *gag* gene sequence (LTR-*gag*) of proviral DNA located within the PCR target region [57]. Sequences were submitted to GenBank with accession numbers PQ423769 through PQ423781. The LTR-*gag* sequences were denoted by the first letter, indicating SRLV genotype according to nRT-PCR (A or B), the two-letter country code of Hungary (HU), and the two-letter herd symbol (e.g., AC), followed by the ordinal number of a goat from which the sequence was obtained (Table S3).

### 2.3. Phylogenetic Analysis

The following reference proviral DNA sequences were selected from GenBank to construct the phylogenetic tree and determine the subtypes to which the Hungarian strains belonged: (1) classical representatives of each genotype—genotype A (subtype A1) Icelandic Visna/Maedi virus Kv1514 (accession no. M10608), genotype B (subtype B1) CAEV-Cork strain (accession no. M33677), genotype C 1GA strain (accession no. AF322109), genotype E Roccaverano strain (E1; accession no. EU293537), and Seui strain (E2; accession no. GQ381130); (2) SRLV strains in which the highest percentage of identity of LTR-*gag* proviral sequences to the Hungarian sequences was detected in the Standard Nucleotide BLAST. Phylogenetic and molecular evolutionary analyses were conducted using the Molecular Evolutionary Genetics Analysis (MEGA) software version 11 [59]. First, the sequences were aligned using the Muscle algorithm with the neighbor-joining (NJ) cluster method [60]. The evolutionary distances were computed using the Tamura-Nei method (d_TH_) [61], including both transitional and transversional substitutions, and assuming the uniform distribution of rates among sites. All positions containing gaps and missing data were eliminated (complete deletion option). The evolutionary distances were expressed in the units of the number of nucleobase substitutions per site and standard errors (±SE) were calculated using the bootstrap method (1000 replicates). The phylogenetic tree was constructed using the NJ method. The confidence level of the topologies (branching orders) was assessed with 1000 bootstrap replicates and presented on the phylogenic tree as the proportion of bootstrap trees for which the same topology was observed (bootstrap support value in %) [62]. A strain was classified to a certain subtype if it differed from the prototypic strain(s) by less than 0.15 nucleobase substitutions per site (≥85% identity) [29,63].

### 2.4. Statistical Analysis

Numerical variables were summarized as the median, interquartile range (IQR), and range and categorical variables as the counts and proportions. The Mann–Whitney U test was used to compare Ct values between individual goats infected with only a single SRLV genotype and to compare DNA concentration between nRT-PCR positive and negative samples. In 7 goats positive for both SRLV genotypes, Ct values were compared between the genotypes using the Wilcoxon signed rank test. The 95% confidence intervals (CI 95%) for proportions were calculated using the Wilson score method [64]. All tests were two-tailed, and a significance level (α) was 0.05. A statistical analysis was performed in TIBCO Statistica 13.3 (TIBCO Software Inc., Palo Alto, CA, USA).

## 3. Results

In total, 135 seropositive adult goats, 8 males and 127 females, were selected for nRT-PCR testing. The number of goats tested in each herd ranged from 4 to 21 goats, with a median (IQR) of 15 (13–16) goats. In one herd (HU-BV), only 6 males were tested as there were no seropositive females. Two other tested males came from two separate herds (HU-BN and HU-BQ) (Table S1).

**Figure 1:**
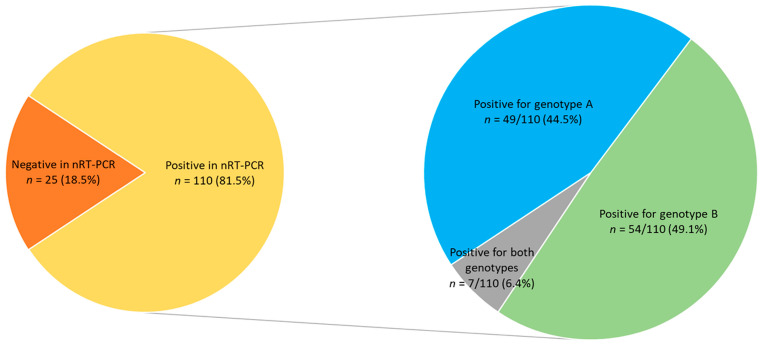
Results of two-stage nested real-time PCR (nRT-PCR) performed on 135 small ruminant lentivirus (SRLV)-seropositive goats from Hungarian herds.

Proviral DNA of SRLV was detected in goats from all 10 tested herds. A single SRLV genotype was detected in 6 herds—genotype A in 3 herds (HU-AC, HU-AL, and HU-BI) and B also in 3 herds (HU-BP, HU-BW, and HU-CA). In 4 herds, mixed infection with genotypes A and B was confirmed. In 2 of these 4 herds (HU-AI and HU-BN) 4 and 3 goats, respectively, positive for both genotypes were also found. In total, 110/135 seropositive goats tested positive in the nRT-PCR (81.5%, CI 95%: 74.1%, 87.1%): 49/110 goats (44.5%, CI 95%: 35.6%, 53.9%) for genotype A, 54 / 110 goats (49.1%, CI 95%: 39.9%, 58.3%) for genotype B, and 7/110 goats (6.4%, CI 95%: 3.1%, 12.6%) for both genotypes (Figure 1).

**Figure 2:**
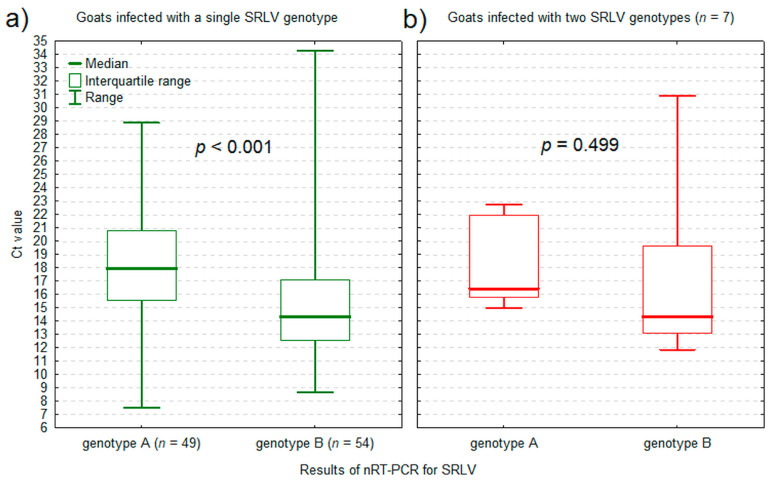
The RT-PCR cycle threshold (Ct) value of the nested real-time PCR (nRT-PCR) for small ruminant lentivirus (SRLV) genotype A and B in goats infected with a single genotype (**a**) and in goats co-infected with genotype A and B (**b**).

The concentration of extracted DNA in the samples ranged from 6.13 to 1438 ng/μL and was significantly higher in 107 nRT-PCR-positive samples (median: 145.5 ng/μL, IQR: 79.5 to 229.3 ng/μL; results for 3 samples missing) than in 25 nRT-PCR-negative samples (median: 66.1 ng/μL, IQR: 39.2 to 120.0 ng/μL; *p* = 0.009). Ct values of the nRT-PCR ranged from 7.5 to 28.9 (median: 18.9, IQR: 15.6 to 20.9) for genotype A and from 8.7 to 34.9 (median: 14.3, IQR: 12.7 to 17.1) for genotype B (Table S3). In goats infected with a single genotype, Ct values of the nRT-PCR for genotype B were significantly lower than Ct values of the nRT-PCR for genotype A (*p* < 0.001) (Figure 2a). In goats infected with both genotypes, there was no significant difference in Ct values between the nRT-PCR for genotypes A and B (*p* = 0.499) (Figure 2b).

PCR products from 14 SRLV strains were submitted for the Sanger dideoxy sequencing—six from herds in which only one SRLV genotype was detected (three of genotype A and three of genotype B) and eight from four herds with mixed infection (four of genotype A and 4 of genotype B). The proviral DNA sequence of SRLV genotype B from one herd with mixed infection (HU-BQ) could not be properly sequenced. Eventually, 13 proviral LTR-*gag* sequences were obtained—seven of genotype A and six of genotype B. They were from 213 to 247 bp-long (median of 234 bp) and covered mostly the LTR region (80.6% to 94.5% of nucleotides in the sequence, median of 83.8%). Analysis by the Standard Nucleotide BLAST showed that all sequences of genotype B were most similar to subtype B1: 4 sequences to the Mexican FESC-752 strain (accession no. HM210570) and two sequences to the Italian SRLV020 strain (accession no. MG554414) with 88%–94% identity. Of 7 sequences of genotype A, four sequences were most similar to the American USMARC-200103515-1 strain from subtype A2 (accession no. MT993899) with 92%–93% identity and 3 sequences were most similar to the Italian SRLV009 strain from subtype A18 (accession no. MG554409) with 87%–89% identity.

In the phylogenetic analysis of LTR-*gag* sequences, in terms of the genotype A, 4 sequences (A-HU-AI46, A-HU-BI26, A-HU-BN34, and A-HU-BV22) were closest to subtype A2. Two other sequences (A-HU-BQ25 and A-HU-AL1) were closest to subtype A18 although their distance from subtype A1 was only slightly larger. On the other hand, sequence A-HU-AC8 was closest to subtype A1 but it was also very close to subtype A18 (Table 1). In terms of genotype B strains, 4 sequences (B-HU-AI16, B-HU-BQ8, B-HU-BW77, and B-HU-CA13) were most similar to the Mexican FESC-752 strain. One sequence (B-HU-BN31) was closest to the Italian SRLV020, and only slightly more distant from the CAEV-Cork, while another sequence (B-HU-BV24) was minimally closer to the CAEV-Cork than to the SRLV020 strain (Table 1).

**Figure 3:**
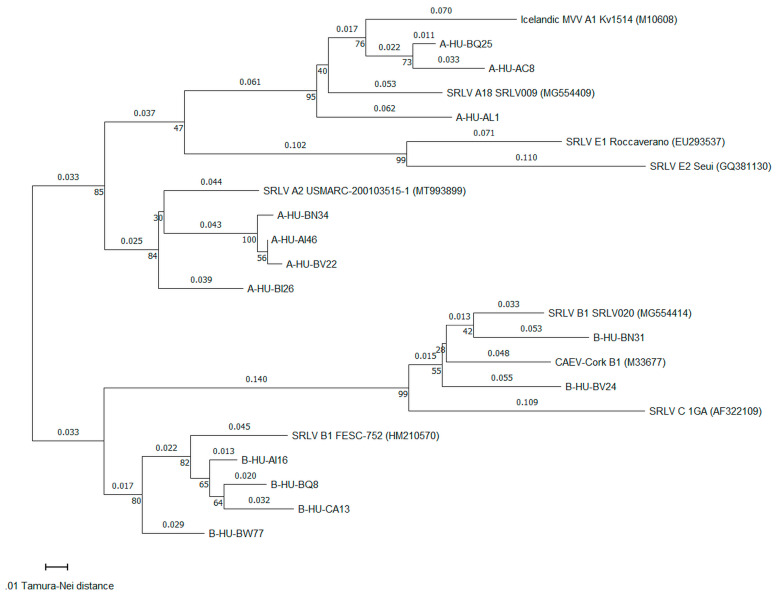
Phylogenetic tree made of 13 long terminal repeat (LTR)-*gag* gene sequences (length of 200-250 bp) from Hungarian small ruminant lentivirus (SRLV) strains and 9 reference SRLV strains, constructed using the neighbor-joining cluster method. Evolutionary distances calculated using Tamura-Nei method are reported above the horizontal branches and bootstrap support values (in %) are presented next to the nodes of the phylogenetic tree.

Generally, 13 Hungarian sequences formed four clusters—two within genotype A and two within genotype B (Figure 3). Four sequences (A-HU-BN34, A-HU-AI46, A-HU-BV22, and A-HU-BI26) clustered around subtype A2 and three sequences (A-HU-AC8, A-HU-AL1, and A-HU-BQ25) clustered around subtypes A1 and A18. Another 4 sequences (B-HU-AI16, B-HU-BQ8, B-HU-CA13, B-HU-BW77) clustered around the Mexican FESC-752 strain from subtype B1 and two sequences (B-HU-BN31 and B-HU-BV24) around the American CAEV-Cork and Italian SRLV020 strain also from subtype B1. Interestingly, the two latter Hungarian sequences were also quite close (d_TH_ = 1.7) to the prototypic 1GA strain of genotype C (Table 2).

There was a considerable heterogeneity of LTR-*gag* sequences within subtype B1—the Mexican FESC-752 strain was distant from the American CAEV-Cork (d_TH_ = 0.297 ± 0.053) and the Italian SRLV020 (d_TH_ = 0.280 ± 0.054) which were in turn very similar to each other (d_TH_ = 0.092 ± 0.026).

## 4. Discussion

Our study shows that both genotypes A and B are present in Hungarian goat herds infected with SRLV. Mixed infections with genotypes A and B are not uncommon at the herd level (4/10 herds) and they also occur at the animal level (7/110 infected goats). SRLV strains of genotype B most likely belong to a classical subtype B1. SRLV strains of genotype A most likely belong to a subtype A2 and also to subtype A1 and/or A18.

The detection of both SRLV genotypes is not surprising. In most countries in which molecular surveys have been carried out, both genotypes were found in goat populations [51,65,66,67,68]. It results from the fact that no interspecies barrier between sheep, goats, and likely some wild ruminants exist, and SRLV readily spreads between small ruminant species [3,4,7]. The nRT-PCR used in our study has been shown to accurately distinguish between genotypes A and B [39,57]. Also, a relative dSe of the nRT-PCR according to serological status was similar in our study to the results obtained by Schaer et al. [57]—81.5% (CI 95%: 74.1%, 87.1%) vs. 75.5% (CI 95%: 68.8%, 81.4%), respectively. This indicates that our results are highly trustworthy.

The limited dSe of nRT-PCR compared to serological tests is a consistent observation in most studies [54,57]. It is likely to result from the scarcity of SRLV-infected monocytes in the blood of SRLV-infected goats. So far, studies have shown that, on average, only approximately 1 out of 10^6^ peripheral blood leukocytes (PBL) harbors the virus and 10^5^ to 5 × 10^7^ PBL are needed to isolate the virus [69]. This is because monocytes are the only leukocytes susceptible to SRLV infection [70,71,72], which can serve as a source of proviral DNA, and they are also one of the rarest leukocyte subclasses in the goat blood [73]. Therefore, the volume of a collected blood sample may affect the dSe of PCR and this may account for slightly higher dSe of the nRT-PCR according to serological status observed in our study in which 10 mL of blood were used to obtain leukocyte buffy coat compared to the study of Schaer et al. [57] in which only 0.75 mL of blood was used. To verify the suspicion that a lower amount of DNA in a PCR-tested sample was associated with negative nRT-PCR results in the seropositive Hungarian goats, we compared the concentration of extracted DNA between the nRT-PCR-positive and negative samples. Even though the nRT-PCR-positive samples contained significantly more DNA, positive results were obtained even in a sample containing as little as 6 ng/μL of DNA. Moreover, our personal observations suggest that repeated measurements of extracted DNA concentration in the same sample differ substantially. Therefore, any conclusions regarding the relationship between DNA concentration and dSe of the nRT-PCR should be drawn very cautiously.

The Ct values obtained in the nRT-PCR for positive samples were significantly lower in goats infected with genotype B, which may suggest higher proviral loads in these goats. Nevertheless, it is crucial to keep in mind that in this study we did not perform a quantitative analysis since no reference samples of known proviral load were used to prepare an appropriate reference curve. In fact, we do not know the efficiency of our nRT-PCR on these samples, and it is possible that the primers and probes may simply be more closely related to sequences of genotype B than A. Therefore, Ct values should only be treated as somehow indicative of possibly higher proviral loads in goats infected with genotype B, historically considered as a “caprine” virus. Further detailed quantitative analysis with the appropriate controls of the proviral load in these animals is warranted to clarify the source of observed differences.

Our study was too small to provide any quantitative estimates of the prevalence of genotypes at any level. However, it was able to detect a given genotype if present in at least 30% of infected goats enrolled in the study. The fact that we detected both genotypes in as many as four of ten tested herds indicates that both genotypes may be equally common in the Hungarian goat population. Moreover, a high proportion of herds with mixed infections also implies intensive movements of goats among herds. Given that the Hungarian goat population is mainly managed according to the extensive and semi-intensive farming systems, between-herd transmissions are likely to be associated with an exchange of male goats between farms before mating seasons. Such a practice is aimed at avoiding inbreeding, and it is typical of extensive farming systems. It has been found to be an important route of CAE transmission in the Polish goat population [74,75].

The phylogenetic analysis and assignment of the Hungarian SRLV strains to certain subtypes must be treated with caution. Our phylogenetic analysis was based on a short proviral DNA sequence (200–250 bp), mainly from the LTR region (roughly 90% of the sequence in this region). Although some studies have employed LTR sequencing in phylogenetic analyses [68,76], the classification of SRLV into subtypes is classically based on sequencing and comparing long fragments of genome including approximately 1.8 kb-long part of *gag* gene and approximately 1.2 kb-long part of *gag-pol* genes [29], rather than of short non-coding terminal fragments. The fact that a high percentage of identity has been observed for the LTR sequences of two strains (tested and reference) does not automatically mean that their protein-coding regions are also close, and they truly belong to the same subtype. Obviously, this is highly likely as mutation rate is higher in the LTR regions than in protein-coding regions of genome [77]. Therefore, if LTR sequences are similar, the strains to which they belong are highly likely to be closely related. However, there are two interesting observations indicating that analyzing LTR regions may be misleading. First is a long evolutionary distance between the prototype CAEV-Cork strain and the Mexican FESC-752 strain although they both belong to the same subtype B1—d_TN_ equals 0.3 while the distance of less than 0.15 is considered as necessary to classify strains to the same subtype. Second is a short evolutionary distance between two Hungarian strains classified into subtype B1 (B-HU-BN31 and B-HU-BV24) and 1GA strain prototypic of genotype C—d_TN_ equals only 0.17. Similar observation was made in previous studies between LTR sequences of genotype C and subtype B3 [68,76]. Obviously, the threshold of 0.15 comes from the study of Shah et al. [29] and applies to long *gag* and *gag-pol* regions.

It was not possible in our study to ultimately decide whether some sequences of SRLV strains of genotype A (A-HU-AC8 and A-HU-BQ25) were more likely to belong to subtype A1 or A18. The evolutionary distances between these strains and prototype strains of subtypes A1 and A18 were very similar. A better candidate seems to be subtype A18 as SRLV strains with a high percentage of identity to this subtype were identified as most common among Croatian SRLV strains tested by Schaer et al. [57] and Croatia is a southern neighbor of Hungary with quite long common border. Moreover, subtype A1 has not been detected in the countries located to the south or east of Hungary, such as Romania [50], Italy [78] or Greece [79]. On the other hand, subtype A1 has been found in Poland [66] which is a country of similar history of purchasing goats from Western Europe [80]. It is not impossible that inconclusive strains are in fact some local recombinants of subtypes A1 and A18, or maybe some other or novel SRLV subtypes as it has been shown in Poland [81] and recently in Greece [82]. Sequencing of long *gag* and *pol* regions or of the whole genome could possibly sort this issue out.

Noteworthy, notation A18 was simultaneously given by two groups of scientists to two different isolates and this fact may be the source of confusion. Olech et al. [81] denoted by A18 the isolates from Polish sheep (only partial *gag* sequences submitted to GenBank in February 2019; accession no. MH790887) while Colitti B., Rosati S., and Bertolotti L. denoted by A18 the Italian (Piedmont region) SRLV009 isolate from goats (whole virus sequence submitted to GenBank in March 2019; accession no. MG554409). In our study, we obviously referred to the Italian SRLV009 as the Polish sequence MH790887 does not contain LTR region.

## 5. Conclusions

In conclusion, our study is the first to show that Hungarian goats are infected by SRLV belonging to both genotypes A and B, and at least two different subtypes of genotype A (A2 and A1 or A18) circulate in this population. SRLV strains of genotype B appear to be more homogenous, all belonging to subtype B1. However, it is a relatively small-scale study based on analyzing only a short part of viral genome, so it is still possible that other SRLV subtypes also circulate in the Hungarian goat population.

## Figures and Tables

**Table 1:** Estimates of evolutionary distances between 13 Hungarian small ruminant lentivirus (SRLV) LTR-*gag* sequences and 9 SRLV reference strains (included as whole-genome sequences) calculated as the number of nucleobase substitutions per site between SRLV sequences (standard errors in parentheses).

SRLV Strain	GenBank Accession Number	Genetic Subtype	Hungarian LTR-*gag* Sequences												
			A-HU-AC8	A-HU-BQ25	A-HU-AL1	A-HU-AI46	A-HU-BI26	A-HU-BN34	A-HU-BV22	B-HU-BN31	B-HU-BV24	B-HU-AI16	B-HU-BQ8	B-HU-BW77	B-HU-CA13
Icelandic MVV Kv1514	M10608	A1	0.105 (0.028)	0.121 (0.031)	0.158 (0.035)	0.224 (0.044)	0.215 (0.043)	0.241 (0.047)	0.233 (0.046)	0.391 (0.068)	0.409 (0.069)	0.335 (0.065)	0.385 (0.075)	0.326 (0.064)	0.372 (0.073)
American CAEV-Cork	M33677	B1	0.409 (0.071)	0.377 (0.065)	0.425 (0.075)	0.392 (0.070)	0.390 (0.070)	0.390 (0.070)	0.392 (0.070)	0.116 (0.032)	0.106 (0.028)	0.281 (0.053)	0.311 (0.058)	0.280 (0.052)	0.281 (0.053)
SRLV 1GA	AF322109	C	0.493 (0.087)	0.471 (0.084)	0.424 (0.073)	0.410 (0.070)	0.421 (0.072)	0.409 (0.070)	0.410 (0.070)	0.172 (0.041)	0.166 (0.038)	0.306 (0.055)	0.337 (0.060)	0.290 (0.055)	0.316 (0.057)
SRLV Roccaverano	EU293537	E1	0.348 (0.064)	0.276 (0.051)	0.340 (0.059)	0.318 (0.059)	0.287 (0.055)	0.316 (0.058)	0.327 (0.060)	0.470 (0.079)	0.417 (0.067)	0.311 (0.056)	0.331 (0.058)	0.339 (0.058)	0.363 (0.064)
SRLV Seui	GQ381130	E2	0.366 (0.068)	0.351 (0.068)	0.315 (0.058)	0.382 (0.071)	0.324 (0.059)	0.379 (0.069)	0.391 (0.072)	0.488 (0.087)	0.445 (0.079)	0.382 (0.066)	0.407 (0.071)	0.317 (0.055)	0.437 (0.081)
SRLV FESC-752	HM210570	B1	0.324 (0.062)	0.311 (0.059)	0.309 (0.057)	0.222 (0.047)	0.204 (0.043)	0.222 (0.046)	0.232 (0.049)	0.311 (0.060)	0.281 (0.051)	0.070 (0.022)	0.077 (0.023)	0.098 (0.025)	0.091 (0.026)
SRLV SRLV020	MG554414	B1	0.387 (0.067)	0.357 (0.062)	0.365 (0.063)	0.414 (0.074)	0.416 (0.078)	0.413 (0.074)	0.414 (0.075)	0.086 (0.026)	0.114 (0.031)	0.300 (0.056)	0.291 (0.056)	0.261 (0.050)	0.282 (0.055)
SRLV USMARC-200103515-1	MT993899	A2	0.213 (0.044)	0.165 (0.036)	0.188 (0.039)	0.085 (0.025)	0.084 (0.025)	0.099 (0.027)	0.093 (0.026)	0.393 (0.072)	0.414 (0.075)	0.207 (0.044)	0.198 (0.043)	0.135 (0.034)	0.224 (0.048)
SRLV SRLV009	MG554409	A18	0.134 (0.033)	0.098 (0.026)	0.112 (0.029)	0.209 (0.043)	0.202 (0.044)	0.225 (0.044)	0.219 (0.045)	0.370 (0.065)	0.367 (0.064)	0.288 (0.057)	0.349 (0.066)	0.285 (0.056)	0.361 (0.069)

Hungarian LTR-*gag* sequences classified into subtype A1/A18 in light blue, classified into subtype A2 in dark blue, and classified into subtype B1 in green. Low evolutionary distances of two strains of subtype B1 from genotype C are highlighted in red.

**Table 2:** Estimates of evolutionary distances between 13 Hungarian small ruminant lentivirus (SRLV) LTR-*gag* sequence.

	A-HU-AC8	A-HU-BQ25	A-HU-AL1	A-HU-AI46	A-HU-BI26	A-HU-BN34	A-HU-BV22	B-HU-BN31	B-HU-BV24	B-HU-AI16	B-HU-BQ8	B-HU-BW77	B-HU-CA13
A-HU-AC8	-	0.044 (0.017)	0.133 (0.031)	0.204 (0.042)	0.221 (0.044)	0.221 (0.043)	0.213 (0.043)	0.420 (0.071)	0.453 (0.076)	0.304 (0.058)	0.362 (0.070)	0.322 (0.062)	0.349 (0.068)
A-HU-BQ25	0.044 (0.017)	-	0.148 (0.033)	0.172 (0.036)	0.197 (0.041)	0.188 (0.038)	0.180 (0.037)	0.389 (0.067)	0.420 (0.071)	0.293 (0.057)	0.346 (0.066)	0.284 (0.056)	0.335 (0.064)
A-HU-AL1	0.133 (0.031)	0.148 (0.033)	-	0.189 (0.040)	0.214 (0.042)	0.205 (0.041)	0.198 (0.041)	0.434 (0.074)	0.397 (0.068)	0.324 (0.061)	0.335 (0.064)	0.292 (0.055)	0.359 (0.068)
A-HU-AI46	0.204 (0.042)	0.172 (0.036)	0.189 (0.040)	-	0.085 (0.026)	0.012 (0.009)	0.006 (0.007)	0.434 (0.084)	0.414 (0.077)	0.190 (0.043)	0.199 (0.044)	0.200 (0.045)	0.208 (0.046)
A-HU-BI26	0.221 (0.044)	0.197 (0.041)	0.214 (0.042)	0.085 (0.026)	-	0.099 (0.028)	0.092 (0.027)	0.438 (0.092)	0.417 (0.079)	0.174 (0.040)	0.191 (0.042)	0.173 (0.040)	0.208 (0.046)
A-HU-BN34	0.221 (0.043)	0.188 (0.038)	0.205 (0.041)	0.012 (0.009)	0.099 (0.028)	-	0.018 (0.011)	0.434 (0.084)	0.414 (0.077)	0.190 (0.042)	0.198 (0.044)	0.199 (0.044)	0.207 (0.045)
A-HU-BV22	0.213 (0.043)	0.180 (0.037)	0.198 (0.041)	0.006 (0.007)	0.092 (0.027)	0.018 (0.011)	-	0.434 (0.084)	0.415 (0.077)	0.199 (0.045)	0.208 (0.046)	0.209 (0.047)	0.217 (0.047)
B-HU-BN31	0.420 (0.071)	0.389 (0.067)	0.434 (0.074)	0.434 (0.084)	0.438 (0.092)	0.434 (0.084)	0.434 (0.084)	-	0.108 (0.029)	0.296 (0.059)	0.314 (0.060)	0.273 (0.053)	0.288 (0.059)
B-HU-BV24	0.453 (0.076)	0.420 (0.071)	0.397 (0.068)	0.414 (0.077)	0.417 (0.079)	0.414 (0.077)	0.415 (0.077)	0.108 (0.029)	-	0.279 (0.051)	0.279 (0.052)	0.262 (0.049)	0.262 (0.050)
B-HU-AI16	0.304 (0.058)	0.293 (0.057)	0.324 (0.061)	0.190 (0.043)	0.174 (0.040)	0.190 (0.042)	0.199 (0.045)	0.296 (0.059)	0.279 (0.051)	-	0.039 (0.016)	0.064 (0.020)	0.052 (0.020)
B-HU-BQ8	0.362 (0.070)	0.346 (0.066)	0.335 (0.064)	0.199 (0.044)	0.191 (0.042)	0.198 (0.044)	0.208 (0.046)	0.314 (0.060)	0.279 (0.052)	0.039 (0.016)	-	0.085 (0.024)	0.052 (0.020)
B-HU-BW77	0.322 (0.062)	0.284 (0.056)	0.292 (0.055)	0.200 (0.045)	0.173 (0.04)	0.199 (0.044)	0.209 (0.047)	0.273 (0.053)	0.262 (0.049)	0.064 (0.020)	0.085 (0.024)	-	0.106 (0.028)
B-HU-CA13	0.349 (0.068)	0.335 (0.064)	0.359 (0.068)	0.208 (0.046)	0.208 (0.046)	0.207 (0.045)	0.217 (0.047)	0.288 (0.059)	0.262 (0.050)	0.052 (0.020)	0.052 (0.020)	0.106 (0.028)	-

Four clusters formed by the Hungarian LTR-*gag* sequences are denoted by colors—dark blue for the cluster around subtype A2, light blue—subtypes A1/A18, dark green—around the American CAEV-Cork and Italian SRLV020 strain from subtype B1, light green—around the Mexican FESC-752 strain from subtype B1.

## Data Availability

All data are available in the Supplementary files: Tables S1 and S3.

## Supplementary Materials

The following supporting information can be downloaded at: https://www.mdpi.com/article/10.3390/pathogens13110939/s1: Table S1. Detailed data of 10 Hungarian herds included in the study; Table S2. Primers used in the two-stage nested real-time PCR (nRT-PCR) for detection of proviral DNA of small ruminant lentivirus (SRLV) and discrimination between genotype A and B (based on Schaer et al. [57]); Table S3. Individual results of the nested real-time PCR (nRT-PCR) for small ruminant lentivirus infection.
