# Supplementary material for: First Molecular Characterization of Small Ruminant Lentiviruses in Hungarian Goat Population
Source: Pathogens. 2024 Oct 29;13(11):939. doi: 10.3390/pathogens13110939 (PMC11597388; doi:10.3390/pathogens13110939)
Supplement: Supplementary file 1 [file pathogens-13-00939-s001.zip › Table S2.pdf]

Table S2. Primers used in the two-stage nested real-time PCR (nRT-PCR) for detection of proviral DNA of small ruminant lentivirus (SRLV) and discrimination between genotype A and B (based on Schaer et al. [57])

Nested Real-Time PCR (nRT-PCR)		Oligonucleotide	Primer sequence	Position in the SRLV genome
First step – classical PCR	Outer primers	SRLV-F1	5′-CGCAGGTGGCGCCCAG-3′	158–173
		SRLV-F2	5′-CGCAGSTGGCGCCCAA-3′	158–173
		SRLV-R1	5′-CCTTCTGTCAGGCGCTCCCC-3′	622–642
		SRLV-R2	5′-CCTTCCGTCAAGGTCTCCTTCC-3′	621–642
		SRLV-R3	5′-CCTTCTGTCAAGGTCTCCTTCCC-3′	620–642
		SRLV-R4	5′-CCTTCTGTCAAGTGCTCCCCTCT-3′	620–642
		SRLV-R5	5′-CCTTCTGTCAGGTGCTCCCCTCT-3′	620–642
Second step – real time PCR (RT-PCR)	Genotype A-specific RT-PCR	RT-A-LTRgag-F	5′-GGGGACGCCTGAAGTRAGGTAA-3′	287–308
		RT-A-LTRgag-R	5′-YTTGAGCTCRGGGTAYCCCTT-3′	517–537
		RT-A-LTRgag-P (probe)	5′-FAM-CTTTGAGCCTTGCKTCGCCATGTCT-TAMRA-3′	486–510
	Genotype B-specific RT-PCR	RT-B-LTRgag-F	5′-CTGRAGGAGTAMGGTAAGTRACTCTGC-3′	324–350
		RT-B-LTRgag-R	5′-TTGATRCATTTKTCSAKCTCAGGATAA-3′	565–591
		RT-B-LTRgag-P (probe)	5′-FAM-CCGGAGACTTGCCTCGCCATGTC-TAMRA-3′	530–552

A – adenine, T – thymine, C – cytosine, G – guanine, R – any purine (A or G), Y – any pyrimidine (C or T), K – G or T, S – G or C, FAM – fluorescein, TAMRA – carboxytetramethylrhodamine, F – forward, R – reverse
